# Blood biomarkers as potential malnutrition screening alternatives among adult patients with cancer on treatment in oncology unit of jimma tertiary hospital: A cross-sectional analysis

**DOI:** 10.1186/s40795-023-00694-0

**Published:** 2023-03-03

**Authors:** Aklesya Enkobahry, Tariku Sime, Kumsa Kene, Tigist Mateos, Sefie Dilnesa, Belay Zawdie

**Affiliations:** 1 Biomedical sciences department, College of Medicine and Health Science, Injibara University, Injibara, Ethiopia; 2 Biomedical Sciences Department, Institute of Health, Jimma University, Jimma, Southwest Ethiopia; 3 Family Medicine Department, Felegehiwot Specialized Hospital, Bahirdar, Ethiopia

**Keywords:** Cancer, Hgb, JMC, Malnutrition, Serum albumin, SGA Tool and TP

## Abstract

**Background:**

Cancer is becoming the most common global public health concern. Early malnutrition detection and treatment in patients with cancer is an important aspect of cancer management. Although Subjective global assessment (SGA) is a gold standard nutritional assessment tool, it is not routinely utilized due to it is tedious and needs patient literacy. Thus, early detection of malnutrition necessitates alternative parameters comparable with SGA. Therefore this study aims to evaluate serum albumin, Total Protein (TP), and Hemoglobin (Hgb) and their correlation with malnutrition in patients with cancer at Jimma Medical Center (JMC).

**Methods:**

Facility based cross-sectional study was conducted from October 15 to December 15, 2021 G.C at JMC among a total of 176 adult patients with cancer selected via a systematic sampling technique. Nutritional status and behavioural data were collected using the SGA tool and a structured questionnaire. Five ml of venous blood was collected and the level of serum albumin, TP and Hgb were measured using Cobas®6000 chemistry analyzer and hematology analyzer UniCel DxH 800. Descriptive statistics, independent t-test, Pearson’s correlation coefficient (r), and logistic regression analysis were implemented for analysis.

**Result:**

From the total of 176 study participants, 69.3% were females and the mean age was 50.1 ± 13.7 years. Based on SGA, 61.4% of the patients were malnourished. There was a significant decrease in the mean level of serum albumin, TP and Hgb in malnourished as compared to well-nourished patients. Serum albumin(r=-0.491), TP(r=-0.270), and Hgb (r=-0.451) had a significant correlation with SGA tool. Stage IV cancer AOR = 4.98 (1.23–20.07), Gastrointestinal (GI) cancer AOR = 3.39(1.29–8.88) and malnutrition AOR = 3.9(1.81–8.4) were significantly associated with hypoalbuminemia. Similarly, age of > 64 years AOR = 6.44(1.55–26.67), GI cancer AOR = 2.92(1.01–6.29) and malnutrition AOR = 3.14(1.43–6.94) were significantly associated with hypoproteinemia; and stage-IV cancer AOR = 3.94(1.11–13.35) and malnutrition AOR = 3.8(1.82–8.2) were significantly associated with low Hgb level.

**Conclusion and recommendation:**

Altered level of serum albumin, TP and Hgb was correlated with the SGA tool of malnutrition. Therefore, it is suggested to be used as an alternative or additional screening tool for prompt detection of malnutrition in adult patients with cancer.

**Supplementary Information:**

The online version contains supplementary material available at 10.1186/s40795-023-00694-0.

## Background

Cancer is the second leading cause of mortality and a global public health concern which is characterized by abnormal cell division [1, 2]. The global evidence in 2018 revealed that the cancer burdens in 2018 was 18.1 million new cases and 9.6 million deaths; and in 2040, it is projected that it will accelerate to 29.5 million new cases and 16.4 million deaths [3]. Even though different cancer types are rampant, breast (11.7%), lung (11.4%), colorectal (10.0%), prostate (7.3%), and stomach (5.6%) cancer are the most common incidence cancer cases [2]. Cancer therapies, surgery, chemotherapy and radiation, are designed to kill or remove cancer cells [4, 5]. Cancer-related metabolic stress, psychological stress, pain and cancer therapies side effect including oral mucositis, constipation, impaired sense of taste and tissue damage causes cancer-related malnutrition, which is a complex metabolic disorder manifested with weight loss, skeletal muscle loss and adipose tissue loss [6–8].

Malnutrition readily apparent in 20–70% of patients with cancer resulting in a poor prognosis, interrupt serial treatment regimen, performance status, extended hospital stay, treatment failure, decreased quality of life, and survival time and lonely it accounts for 10–20% of death [9–11]. A study in Malaysia and Nairobi, Kenya, showed that 43.5% and 31% of the adult patients with cancer are malnourished [12, 13]. Particularly in Ethiopia, 5.8% of total death reported from national mortality is due to cancer [14]. Two-thirds of cancer deaths are due to breast cancer (31.4%), cervical cancer (14.3%) and ovarian cancer (6.3%) [14]. A study in central Ethiopia showed that malnutrition accounts for 58.4% of patients with cancer [15].

Malnutrition has been commonly diagnosed through Body Mass Index (BMI), which leads us to misdiagnosis and underestimate cancer-related malnutrition [16, 17]. This is due to; BMI measures the whole body which does not differentiate between muscle and fat mass. Moreover, it is also affected by the age, sex, edema, height of an individual and measurement error [18]. Hence, there is still a problem in the diagnosis of malnutrition among patients with cancer [19]. Nowadays, Subjective Global Assessment (SGA) is the most common, effective, reliable nutritional assessment tool [20] and is validated with high sensitivity (96%), good specificity (83%) and high inter-rater agreement in patients with cancer [20, 21]. However, SGA is valid, not common in routine clinical service of developing country hospital diagnostic setting due to having multiple items with its score which is time-consuming, tedious, requires more professional human resources, requires training, relies on patient literacy and not familiar with oncology nurses despite applying in different research purposes. Therefore, in developing countries like Ethiopia, diagnosis of malnutrition falls under question. Furthermore, it needs alternative objective biochemical parameters that facilitate early diagnosis of malnutrition and intervention among patients with cancer. Furthermore, even though studies on malnutrition among patients with cancer were conducted in Ethiopia the existing literature was inconsistent with each other and did not address the problem of routine diagnostic modalities.

Albumin is the most abundant circulating acute phase protein produced by liver hepatocytes and a significant antioxidant [22, 23]. In cancer and cancer-related malnutrition, high production of cytokines such as TNF-α, IL (interleukin) -2, and IL-6 cause metabolic disruption which inhibits albumin gene expression and causes vascular permeability. Moreover, nutrient deprivation decreases albumin gene expression and synthesis [24, 25]. According to a study, low albumin levels are directly associated with poor prognosis and survival of breast patients with cancer [26–28]. According to various studies malnutrition is directly associated with serum albumin [29–32]. Serum total protein is mainly composed of albumin and globulin. In patients with cancer, malnutrition is associated with inflammatory mediators by tumors resulting in changes in liver metabolism and TP level [33]. Protein synthesis in the human body is regulated by nutrient-sensing pathways, mechanistic target of rapamycin (m TOR) with optimum nutrient unless in the nutrient-limited environment leading to low total serum protein [34]. In cancer-related malnutrition, metabolic alteration leads to skeletal muscle loss resulting in the degradation of extracellular protein as an amino acid source for tissue protein synthesis [35].

As cancer cells accumulate, pro-inflammatory cytokines cause hemolysis and disrupt glucose metabolism leading to low Hgb levels [24, 36]. This is because of the interdependence between heme and glucose metabolism [37]. Furthermore, an increase in hepatic production of IL-6-induced hepcidin which causes the degradation of cellular ferroportin ultimately results in limited iron access for heme synthesis [36]. A study done in Palestine showed that 24% of the participants had low Hgb levels and 14% had low serum albumin levels [6]. Evidence showed that in patients with cancer’ age, nutritional status, stage and type of cancer are factors associated with albumin, TP and Hgb level [13, 38–44]. This study aimed to evaluate blood biomarkers (serum albumin, TP and Hgb) and their correlation with malnutrition and to identify factors associated with blood biomarkers alteration among adult patients with cancer on treatment follow up.

## Methods and materials

### Study design and setting

A facility based cross-sectional study design was employed from October 15 to December 15, 2021, at the adult oncology unit of Jimma Medical Center (JMC).

### Study participants

All solid tumor patients with cancer ≥ 18 years of age on treatment follow up at JMC during the study period were included in the study. Through reviewing patient’s medical records, adult patients with cancer who are critically ill and unable to respond; with known renal and liver failure, malignancy of liver and kidney, severe anemia, hematologic disorders, Hx of blood transfusion 3 months prior to sample collection, recent trauma, recent burn, physically deformity (kyphosis and scoliosis), DM, HIV/AIDS and cardiac illnesses including chronic heart failure, ischemic heart disease, and hypertensive heart disease were excluded.

### Sample size and sampling techniques

Sample size was determined based on single population proportion formula, we got 160. Finally by adding a 10% non-response rate [45], the total sample size was 176. The study participants were selected by systematic random sampling techniques with sampling interval of k **~** 3(600/176 = 3.4) to select study units from daily coming adult patients with cancer on treatment.

### Data collection procedures and tools

Data were collected by two oncology unit nurse and laboratory technologists using questionnaires, anthropometric measurements, record reviews, and blood sample laboratory test analysis.

### Questionnaires

Data was collected using WHO STEPS Questionnaire [46] adapted to the local context based on the study objectives, Dietary Diversity Score (DDS), FAO [47], and SGA tool.

### Subjective global assessment (SGA) tool

The instrument for data collection of nutritional status among adult patients with cancer was SGA tool which is adapt by Desky [48] and translated and validated in Amharic [49] and employed in Ethiopian patients with cancer [50]. The modified SGA tool adopted from the study in India [51]. SGA contains medical history and physical examination and classifies the patient as: A (well-nourished), B (moderately malnourished), C (severely malnourished) [20]. SGA assessment which scores “B + C” indicates malnutrition [63]. Based on the score SGA < 17 are well-nourished and SGA ≥ 17 are malnourished [20].

### Anthropometrics measurements

The height of the study participants were measured to the nearest 0.1 cm using a stadiometer (Seca Germany) and weight was measured using a digital weight scale to the nearest 0.1 kg (kg). Then, BMI was calculated as weight in kg divided by height in square meters and interpreted as underweight (< 18.5 kg/m^2^), normal weight (18.5–24.9 kg/m^2^), overweight and obese(> 24.9 kg/m^2^) [52].

### Biochemical tests and analysis

After informed consent was taken, the interview and a detailed review of the medical record were done. Then, using aseptic techniques about five milliliters of venous blood samples was drawn by oncology unit nurses from eligible patients with cancer through sterile syringe. Accordingly, 2.5 ml of blood sample was dispensed to a Serum Separator (SS) tube for analysis of serum albumin and TP; and 2.5 ml of whole blood was dispensed to the Ethylene-Diamine-Tetra-Acetic acid (EDTA) tube for analysis of Hgb. The whole blood in the EDTA tube mixing gently to prevent against hemolysis and clot formation then sent to the hematology laboratory of JMC within 30 min of sample collection to determine the Hgb level. Hgb was analyzed by a fully automated hematology Analyzer, UniCel DxH 800 (Beckman Coulter, USA) in hematology unit of JMC by qualified laboratory professional with the assistance of principal investigator (PI). Correspondingly, a blood sample from the SS tube allowed to stand for 30 min at room temperature, to allow complete clotting and clot retraction, then centrifuge the sample at 4000 rpm for 10 min to separate the serum from the whole blood and keep at − 20 °C in the refrigerator till analysis. Determination of serum albumin and TP was performed by qualified laboratory professional with the assistance of PI using an automated Cobas® 6000 chemistry analyzer (Roche diagnostic, Germany) in the clinical chemistry unit, JMC. Study participants results were recorded using laboratory result registration form. Even though blood encompasses numerous biomarkers [22]. For this study purpose it includes albumin and TP in serum and, Hgb in whole blood. The expected normal value for serum albumin, TP, and Hgb were 3.5–5.2 g/dl, 6.6–8.7 g/dL and 12-16 g/dL in adult men and 13–16 in adult women. Below this expected normal values were defined as hypo(Hypoalbuminemia; serum albumin level < 3.5 g/dl, Hypoproteinemia; TP level < 6.6 g/dL and low hgb level, if it is < 13 g/dL in adult’s men and < 12 g/dL in adult women [65, 66].

### Data analysis procedures and tools

Data were entered into Epi-Data version 4.6 and exported to SPSS version 25 for analysis. The data were cleaned through running frequencies and checked for normality and fulfillment of assumptions using histogram and boxplots. Descriptive statistics frequency distributions tables, graphs, means, and standard deviation were employed to describe the findings. An independent t-test analysis was used to compare the mean of blood biomarkers (Serum albumin, TP, and Hgb) between the malnourished and well-nourished groups. Pearson’s correlation coefficient was.

used to correlate the level of blood biomarkers with nutritional status. The bivariate analysis was employed to explore the association between dependent variables (blood biomarkers), and the socio-demographic, behavioral, cancer and their treatment-related factors and Nutritional status. Those variables with a p-value < 0.25 were taken as a candidate for multivariable analysis, in which the confounders were controlled and adjusted to the odds ratio(AOR) with 95% confidence interval (CI) to express the strength of the association between blood biomarkers and the associated factors with statistical significance of p-value < 0.05.

### Data quality management

A one-day training of the contents of the questionnaires ,how to approach study subjects, each item included in questionnaire, the sample collection, biological sample handling, and ethical conduct of human research by medical oncologist and PI. Regular follow up and supervision was made at each phase of the study. Data were collected through local language and re-translated back to English to check the consistency. To ensure quality, the collected data were checked out for completeness, accuracy, and clarity by two data collection supervisors and the principal investigator. Appropriateness of methodologies and excellence of equipment and reagents for the intended study were intimately monitored by experts and principal investigator. All the sample taking procedures, handling, processing and analysis were carried out by strictly following standard operational procedures (SOPs). Internal quality control procedures were implemented for all laboratory procedures. All the reagents were checked for their expiry date and all the instruments were calibrated every day by running quality control samples before the actual sample test according to the manufacture’s recommendation.

## Results

### Sociodemographic, behavioral and clinical characteristics

A total of 176 adult patients with cancer were enrolled in the study. More than two third (69.3%) of the participants were females. The Mean ± SD of age of the respondents was 50.1 ± 13.7 years and 52.3% of them were between 35 and 54 years of age. Regarding with behavioral practices only 8.5% were current alcohol user, 32.9% chew khat, 1.7% smoke cigarette and 83.5% are not performing regular physical exercise as per WHO recommendation. Concerning the type of cancer, 33.52% had breast cancer, 18.75% cervical cancer, 9.09% esophageal cancer, 9.09% ovarian cancer and 7.95% colorectal cancer, bone and soft tissue cancer 7.39%, 4.55% pancreatic cancer, 4.55% Prostate cancer and others 2.84%. The proportion of different stages among respondents diagnosed with cancer was 10.2% stage I, 18.8% stage II, 29.5%, stage III, and 41.5% stage IV. Concerning the type of treatment, 76.7% of the study participants were on chemotherapy, 18.2% were on chemo radiotherapy and 5.1% had surgical treatment. Regarding BMI, 42.6%, were categorized under underweight (< 18.5Kg/m2), 53.4% were normal weight (18.5–24.9 Kg/m2) and 4% were overweight and obese (> 25 Kg/m2) (Table 1).

**Table 1:** Socio demographic, behavioral and clinical characteristics of adult patients with cancer on treatment follow up at JMC, Jimma, Southwest Ethiopia 2021, (n = 176)

Variables	Categories	Frequency (n)	Percent (%)
Sex	Male Female	54 122	30.7 69.3
Age (year)	18–34 35–54 55–64 > 64	27 92 32 25	15.3 52.3 18.2 14.2
	Mean(± SD)	50.1 ± 13.7	
Alcohol use	No Yes	161 15	91.5 8.5
Smoking cigarettes	No Yes	173 3	98.3 1.7
Chat chewing	No Yes	118 58	67.04 32.9
Performing regular physical exercise	No Yes	147 29	83.5 16.5
Family history of cancer	No Yes	144 32	81.8 18.2
Type of cancer	Breast cancer Cervical cancer Esophageal cancer Ovarian cancer Colorectal cancer Bone and soft tissue cancer Pancreatic cancer Prostate cancer Gastric cancer Others	59 33 16 16 14 13 8 8 4 5	33.52 18.75 9.09 9.09 7.95 7.39 4.55 4.55 2.27 2.84
Stage of cancer	Stage I Stage II Stage III Stage IV	18 33 52 73	10.2 18.8 29.5 41.5
Types of cancer treatment	Chemotherapy Chemo radiotherapy Surgery	135 32 9	76.7 18.2 5.1
Loss of appetite	No Yes	39 137	22.2 77.8
Nausea	No Yes	50 126	28.4 71.6
Vomiting	No Yes	84 92	47.7 52.3
BMI (Kg/m^2^ )	< 18.5	75	42.6
	18.5–24.9	94	53.4
	≥ 25	7	4
	Mean(± SD) 19.23 ± 3.42		

*others: includes (head and neck, lung, oral, brain tumor)

### Prevalence of malnutrition among adult patients with cancer

According to SGA tool, malnutrition accounts 61.4% are malnourished (14% severe malnourished and 47.4% are moderately malnourished).

### Blood biomarkers (serum albumin, total protein and hemoglobin) alteration

The magnitude of hypoalbuminemia, hypoproteinemia, low Hgb level was 49.4%, 34.1%, and 50% respectively. Hypoalbuminemia, hypoproteinemia and low Hgb level were more highly distributed in females than male (Table 2).

**Table 2:** Level of serum albumin, total protein and hemoglobin based on sex among adult patients with cancer on treatment follow up at JMC, Jimma, Southwest Ethiopia, 2021(n = 176)

Variables	Category	Sex			Total N = 176 N (%)
		Male = 54 N (%)		Female = 122 N (%)	
Hypoalbuminemia	No Yes	20(11.4) 34(19.3)		69(39.2) 53(30.1)	89(50.6) **87(49.4)**
	Mean(± SD) 3.29 ± 0.67				
Hypoproteinemia	No Yes	29(16.5) 25(14.2)		87(49.4) 35(19.9)	116(65.9) **60 (34.1)**
	Mean(± SD) 6.71 ± 0.99				
Low hemoglobin level	No Yes		22(12.5 ) 32(18.2)	66(37.5) 56(31.8)	88(50 ) **88 (50)**
	Mean(± SD) 11.98 ± 2.18				

*SD: standard deviation*

### Blood biomarker (serum albumin, total protein, and Hemoglobin) and malnutrition

The total number of 176 adult patients with cancer was categorized into well-nourished (38.6%) and malnourished (61.4%) based on SGA tool. There was statistically significant decreased mean level of serum albumin, Hgb and TP in malnourished as compared with well-nourished patients with cancer (Table 3).

**Table 3:** Comparison of mean level of serum albumin, TP and Hgb between well-nourished and malnourished by independent sample t-test among adult patients with cancer on treatment follow up at JMC, Jimma, Southwest Ethiopia (n = 176), 2021

Blood biomarkers	Total AC	Well-nourished (n = 68(38.6%))	Malnourished (n = 108 (61.4%))	*p-value*
	Mean ± SD	Mean ± SD	Mean ± SD	
Serum albumin(g/dl)	3.29 ± 0.67	3.71 ± 0.58	3.03 ± 0.58	*0.000* *
TP (g/dl)	6.71 ± 0.99	7.01 ± 1.01	6.52 ± 0.94	0.002*
Hgb (g/dl)	11.98 ± 2.18	13.26 ± 1.51	11.18+/-2.16	0.000*

Note: * statistically significant at p < 0.05; AC- adult cancer SD: Standard deviation.

### Correlation of Blood Biomarkers (Serum Albumin, Total Protein, and Hemoglobin) Level and SGA score of malnutrition

The bivariate Pearson’s product-moment correlation analysis was employed. The mean level of serum albumin, TP, and Hgb had a negative statistically significant linear correlation with mean level of SGA score in adult patients with cancer. Furthermore, BMI (r= -0.116, p < *0.002)* had a weak significant correlation with SGA score (Table 4).

**Table 4:** Pearson’s correlation analysis between Serum albumin, TP, Hgb and SGA score in adult patients with cancer on treatment follow up in JMC, Jimma, Southwest Ethiopia, 2021 (n = 176)

SGA score(in number)		
Parameters	Pearson’s correlation (r)	*P-value*
Serum albumin (g/dl)	-0.491	*< 0.001*
Tp(g/dl)	-0.270	*< 0.001*
Hgb(g/dl )	-0.451	*< 0.001*
BMI(kg/m^2^ )	-0.116	*0.002*

Note: correlation at p-value < 0.05 is considered statistically significant

### Factors associated with blood biomarkers alteration (serum albumin, total protein, and hemoglobin) level

In the bivariate logistic regression analysis, the candidate variables with p-value of less than 0.25 were selected for final model. Accordingly, sex, loss of appetite, change in weight, SGA score malnutrition, type of cancer, and stage of cancer were a candidadate variable of hypoalbuminemia for multivariable analysis. After confounding variables were controlled, multivariable logistic regression analysis revealed variables, stage IV cancer (AOR = 4.98(1.23–20.07)), GI cancer AOR = (3.39(1.29–8.88)) and malnutrition (AOR = 3.9(1.81–8.4)) with p-value less than 0.05 were significantly associated with hypoalbuminemia at 95% CI with respective AOR. Similarly, six variables (age, sex, and loss of appetite, SGA score malnutrition, type of cancer and stage of cancer) were a candidate for multivariable analysis. After confounding variables were controlled, multivariable logistic regression analysis revealed variables. Age > 64 years (AOR = 6.44(1.55–26.67)), GI cancer (AOR = 2.92(1.01–6.29)), malnutrition (AOR = 3.14(1.43–6.94)) in pateints with cancers were significantly associated with hypoproteinemia at p-value less than 0.05 with 95% CI and respective AOR. Accordingly about seven variables (age, sex, change in weight, nutritional status, type of cancer, stage of cancer, and type of cancer treatment) were identified as the expected factors associated with the development of low Hgb level with 95% CI. Further multivariable analysis was used to identify the main predictor variabels. Stage IV (AOR = 3.94(1.11–13.35)) and malnutrition (AOR = 3.8 (1.82–8.2)) were significantly associated with low Hgb level at p-value less than 0.05 was significant at 95% CI with respective AOR (Table 5).

**Table 5:** Multivariable analysis of factors associated with hypoalbuminemia, hypoproteinemia and low Hgb level among adult patients with cancer on treatment follow up at JMC, Jimma, Southwest Ethiopia, 2021 (n = 176)

Variables	Categories	Hypoalbuminemia	AOR & 95%CI	*P-value*
		Yes N (%)		
Sex	Male	34(63)	1	*0.307*
	Female	53(43.4)	0.62(0.24–1.56)	
Loss of appetite	No	13(33.3)	1	*0.359*
	Yes	74(54)	0.62(0.23–1.71)	
change in weight	Weight gain or No change	5(23.8)	1	
	Moderate weight loss	31(41.3)	1.89(0.51–7.03)	*0.338*
	Severe weight loss	51(63.7)	2.27(0.58–9.02)	*0.241*
Nutritional status	Well-nourished	16(23.5)	1	
	Malnourished	71(65.7 )	**3.9(1.81–8.4)**	0.001**
Type of cancer	Breast cancer	23(39 )	1	
	Genitourinary cancer	25(43.9)	1.15(0.50–2.64)	*0.727*
	Gastrointestinal cancer	31(73.8)	**3.39(1.29–8.88)**	0.013**
	Others	8(44.4 )	1.71(0.51–5.68)	*0.384*
Stage of cancer	Stage I	4(22.2)	1	
	Stage II	9(27.3)	2.09(0.45–9.74)	*0.347*
	Stage III	27(51.9 )	3.64(0.87–15.18)	*0.076*
	Stage IV	47(64.4)	**4.98(1.23–20.07)**	0.024**
		**Hypoproteinemia**	**AOR & 95%CI**	***p-value***
		**Yes N (%)**		
Age in year	18–34	4(14.8)	1	
	35–54	30(32.6)	2.88(0.86–9.60)	*0.084*
	55–64	13(40.6)	3.62(0.94–13.99)	*0.061*
	> 64	13(52)	**6.44(1.55–26.67)**	0.01**
Sex	Male	25(46.3)	1	*0.843*
	Female	35(28.7)	0.91(0.34–2.39)	
Loss of appetite	No	9(18)	1	*0.22*
	Yes	51(40.5)	2.01(0.67–6.08)	
Nutritional status	Well-nourished	12(17.6 )	1	0.004**
	Malnourished	48(44.4)	**3.14(1.43–6.94)**	
Type of cancer	Breast cancer	16(27.15 )	1	
	Genitourinary cancer	15(26.35 )	0.80(0.34–1.91)	*0.623*
	Gastrointestinal cancer	25(59.5)	**2.92(1.01–6.29)**	0.048**
	Others	4(22.2)	0.82(0.22–3.09)	*0.769*
Stage of cancer	Stage I	5(27.8 )	1	
	Stage II	7(21.2 )	0.85(0.19–3.76)	*0.828*
	Stage III	16(30.8)	0.93(0.24–3.68)	*0.923*
	Stage IV	32(43.8 )	1.28(0.35–4.76)	*0.709*
		**Low Hgb level**	**AOR & 95%CI**	***P-value***
		**Yes N (%)**		
Age	18–34	11(40.7)	1	
	35–54	43(46.7)	1(0.35–2.82)	*0.985*
	55–64	18(56.3 )	1.34(0.39–4.57)	*0.637*
	> 64	16(64.0 )	3.29(0.82–13.10)	*0.091*
Sex	Male	32(59.3 )	1	
	Female	56(45.9)	0.77(0.33–1.77)	*0.541*
Change In Weight	Weight gain or No change	4(19.0 )	1	
	Moderate weight loss	32( 42.7 )	2.65(0.63–11.14)	*0.182*
	Severe weight loss	52(65 )	3.72(0.802–17.32)	*0.093*
Nutritional status	Well-nourished	16(23.5)	1	0.000**
	Malnourished	72(66.7)	**3.8(1.82–8.2)**	
Type of cancer	Breast cancer	26(44.1)	1	
	Genitourinary cancer	27(47.4)	0.78(0.3–1.99)	*0.616*
	Gastrointestinal cancer	28(66.7)	1.05(0.31–3.59)	*0.930*
	Other	6(38.9)	0.57(0.12–2.71)	*0.482*
Stage of cancer	Stage I	5(27.8)	1	
	Stage II	3(9.1)	0.26(0.051-`1.37)	*0.105*
	Stage III	28(53.8)	2.08(0.60–7.15)	*0.245*
	Stage IV	52(71.2 )	**3.94 (1.11–13.35)**	0.027**
Type of cancer treatment	Chemotherapy	63(46.7)	1	
	chemo radiotherapy	22(68.8 )	2.35(0.883–6.26)	*0.087*
	surgery	3(33.3)	1.91(0.29–12.36)	*0.493*

Key: **: - variables which are significant at p-value less than 0.05 1 = reference category *AOR: Adjusted odds ratio*

## Discussion

Globally, cancer case and complication is on increasing. Moreover, malnutrition is becoming one of the most common complications of patients with cancer due to systemic effect of the disease and side effects of cancer therapy including oral mucositis, constipation, impaired sense of taste and tissue damage. Hence; early screening of malnutrition is a corner stone in cancer management. SGA as a gold standard in routine service, but the implementation is rare. Therefore, this study aims to evaluate serum albumin, TP and Hgb derangements correlated with SGA tool as an alternative diagnostic modality for early and better diagnosis of malnutrition. In the current study, a total of 176 adult patients with cancer were grouped into malnourished and well-nourished based on SGA nutritional assessment tool validated and employed in patients with cancer [15, 20, 51].

In the current study, from 176 adult patients with cancer, more than two-thirds (69.3%) of the patients were female and 52.3% of them were between 35 and 54 years of age (Table 1). This finding was in harmony with study conducted at TASH, Addis Ababa. This high female adult cancer patient finding might be explained by increased on cancer screening and frequent health facility visits during child birth and pregnancy. Moreover, female can develop cancer related to their reproductive organs. Furthermore, working age group being affected consequently might have influence on economy of the country. From the study participants, stages of cancer were distributed as stage I 18(10.2%), stage II 33(18.8%), stage III 52 (29.5%) and stage IV 73(41.5%) (Table 1). The high percentage of an advanced stage IV finding was consistent with study done in Santamaria, Brazil stage IV (43%) [30]. This high percentage of advanced stage (41.5%) of cancer may be explained due to low awareness on clinical manifestations of cancer, and use of traditional remedies [53, 54].

Our study found that about 49.4% was hypoalbuminemic. The result of hypoalbuminemia in patients with cancer might be due to cancer patient, pro-inflammatory cytokines (TNF-α, IL-2, and IL-6) induced positive acute phase reactant synthesis compete for nutrient in liver leads to decreases in serum albumin production [55]. Another explanation could be highly proliferating cancer cells increased uptake of serum albumin through induce Albumin Binding Proteins (ABP) [56]. But hypoalbuminemia prevalence in this study was high as compared to findings in Ivory coast(13.5%) [57], Palestine(14%) [6] and Malaysia(33%) [13]. The possible justification for discrepancy with study done Ivory coast (53 sample), Palestine (100 sample) and Malaysia (100 sample) might be due to relatively small sample size. Similarly, in the current study magnitude of hypoalbuminemia (49.4%) was higher than study done in Zimbabwe (28.6%). This could be due to relatively small sample size (63 sample), study population difference (on radiotherapy), and limited to few types of cancer (breast, cervical and prostate) [58]. In contrary to this, in the current study, lower prevalence of hypoalbuminemia than the study done in University of Pelotas, Brazil (68.9%) with relatively small sample size (74 sample size) [59].

In this study, there was statstically significant negative correlation(r=-0.491, P < 0.001) between serum albumin and SGA score level (Table 4). The finding is in harmony with the studies conducted at Nigeria [29] and Santamaria, Brazil [30] and Greek [42] reported a significantly low level of serum albumin in patients with cancer with malnutrition. Even though there is no evidence in contrary to the current finding in regards of all cancer types; there was evidence with restricted types of cancer. The study conducted in China reported no significant correlation between serum albumin and malnutrition in patients with cancer. This discrepancy might be due to study population limited with early stage and single type of cancer(esophageal cancer) [60].

In the current study, mean level of serum albumin was low in patients with malnutrition (3.03 ± 0.58 g/dl) as compared to well-nourished (3.71 ± 0.58 g/dl) adult patients with cancer, and the difference was statistically significant (Table 3). The finding of this study is consistent with study done in Santamaria, Brazil [30]. The possible explanation could be in cancer related malnutrition, nutrient deprivation and inflammation, downregulates serum albumin gene expression leading to inhibition of synthesis [24]. Furthermore, possible explanation could be since plasma antioxidant, serum albumin, scavenges high free radical level of oxidative stress in cancer related malnutrition leading to serum albumin depletion [24, 61]. Another possible justification could be, in malnutrition associated glucose metabolic alteration leads to insulin resistance [62], which inhibits serum albumin gene expression. A study done in China which opposes the current finding reported no significant mean difference of serum albumin level between malnourished and well-nourished patients [60]. The difference could be due to their study population restriction to chemo radiotherapy treated esophageal cancer before and after radiation therapy.

In the current finding, malnourished were 4 times more likely to have hypoalbuminemia than well-nourished (Table 5). This study is supported with meta-analysis of hypoalbuminemia as nutritional marker in patients with cancer [38]. The finding also supported with the study conducted in Bari, Italy verified association of hypoalbuminemia with malnutrition among restricted study population of colorectal patients with cancer [39]. The possible justification for this could be due to cancer related malnutrition (CRM) associated inflammatory mediators TNF-α, IL-2,and IL-6 inhibits serum albumin gene expression, enhance vascular permeability of plasma serum albumin clearance, and degradation of serum albumin to generate amino acid for tissue protein synthesis and low protein diet decreases serum albumin level [24].

This study finding shows that Being GI patients with cancer were 3 times more likely to have hypoalbuminemia than breast patients with cancer (Table 5). The finding is supported with study done in Iran serum albumin level is significantly lower in GI patients with cancer than non-GI patients with cancer [63]. Also, advanced stage IV adult patients with cancer (64.4%) had 5 times more likely develop hypoalbuminemia than patients on stage I (Table 5). The finding of this study supported with study done in Malaysia(47.7%) reported stage IV cancer was associated with hypo albuminuria [13]. This is could be due to advanced stage cancer cells increased uptake of serum albumin [56]. But our finding is contrary with study done in Ivory coast, which verify no association between serum albumin level and stage of cancer [57]. This discrepancy with Ivory Coast study may be due to small number of metastatic stage of cancer (12 patients), relatively small sample size (53 sample).

In the current study magnitude of hypoproteinemia was 34.1%. This hypoproteinemia may be due to poor nutritional status, increases degradation of protein for tissue protein synthesis and its antioxidant role. The finding of our study is very close to study done in Algeria (31.1%) [64] with colorectal cancer restricted study participant. In contrary, the study finding magnitude of hypoproteinemia is higher than study done in Zimbabwe (4.7%) [58]. This is due to relatively small sample size (63 samples), study population limitation (on radiotherapy treatment only), and restricted to type of cancer (breast cancer, cervical cancer and prostate cancer).

A statistically significant negative weak correlation (r=-270, p < 0.001) was also observed between TP level and SGA score (Table 4). This is consistent with study done in Greek among oncology patients [42]. In the present study, mean level of TP had statistically significant difference between malnourished (6.52 ± 0.94 g/dl) and well-nourished (7.01 ± 1.01 g/dl) adult patients with cancer (Table 3). This study finding is in line with study done in Greek among oncology patients [42]. In this study, malnutrition was also found significantly associated with hypoproteinemia. Participants who are malnourished (44.4%) were 3 times more likely to have hypoproteinemia than well-nourished (17.6%) adult patients with cancer. It could be due to nutrient deprivation alter protein hemostasis by inhibiting protein synthesis [34].

This study finding showed that adult patients with cancer of age > 64 years was 6 times more likely to have hypoproteinemia than 18–36 years patients with cancer (Table 5). The finding of this study supported with a study conducted among adult patients with cancer in Athens, Greece indicated that age was the predictors of post-operative hypoproteinemia [40]. The possible reason for hypoproteinemia could be, as age progresses, inflammation increases and hepatocyte compromised in number, volume and functions are results in decreased protein synthesis. In contrary, the study done in Zimbabwe shows that no significant difference of hypoproteinemia in different age group [58]. This discrepancy may be due to difference in patient’s age cut-off point to classify age group and previous study was concerned on few cancer types. Patients with GI cancer were 3 times more likely develop hypoproteinemia than breast patients with cancer (Table 5). The possible explanation could be, in GI cancer increase in mucosal permeability caused by inflammation, leading to excessive leakage of serum protein to the gut and reduce its reabsorption leads to hypoproteinemia [65].

In the current study, the magnitude of low Hgb level in patients with cancer was 50%. It could be related to cancer treatment or disease. This magnitude is higher when compared to finding in Palestine (24%) [6], Addis Ababa, Ethiopia (23%) [66], and Saudi Arabia (44.1%) [67]. In contrary to it is very lower as compared to finding in Tanzania(86%) [68], this inconsistencies could be due to study population restricted to radiotherapy patients, and include blood transfused patients (21.3%) in previous study. A significant inverse correlation (r=-0.451, p < 0.001) was also observed between Hgb level and SGA score (Table 4). The finding is supported with study conducted in Jordan among colorectal patients with cancer with study population restriction [69].

This study also found that the mean level of Hgb was significantly decreased in malnourished patients (13.26 ± 1.51 g/dl) as compared to well-nourished (11.18 ± 2.16 g/dl) adult patients with cancer (Table 3). The possible explanation could be in cancer related malnutrition, ROS and IL-6, induces hepcidin, which degrade ferroportin and halt the uptake of iron from small intestine and macrophage leading to unavailability of iron for heme synthesis [36]. Another potential explanation for this might be due to disruption of glucose metabolism contributes to low Hgb levels. This is because the Krebs cycle affects the availability of substrate for heme synthesis [37]. Furthermore, this finding is in parallel with study done in Italy [39]. In contrary to the current finding, study done in Greek revealed that there is statically insignificant differences in mean level of Hgb between malnourished and well-nourished according to SGA [42]. The discrepancy could be due to relatively small sample size (88 patients).

Participants who are malnourished were 3.8 times more likely to have low Hgb level than well-nourished adult patients with cancer (Table 5). This is supported with a study done in Italy [39]. This may be due to in malnourished patients with cancer tumor cell-mediated cytokines such as IL-1, Il-6, and (TNF-α) cause hemolysis, lowering Hgb levels [37]. In the current study advanced stage IV patients with cancer were 3.9 times more likely to have low Hgb than other stage I patients with cancer (Table 5). This finding is supported with study done in Cagliari, Italy which elucidate patients with advanced stage of cancer had low Hgb level [41]. The possible explanation could be as stage of cancer more advanced; tumor cells number and nutrient requirement increased which leads to low Hgb level. Furthermore, in advanced cancer, the increased new blood vessels formation requires blood cell may decreases Hgb level [70]. Similarly, the current study finding is supported with study done in Addis Ababa being stage IV patients with cancer are risky than stage I [53, 66].

### Strength and Limitations

Due to the study was carried on a single cancer center; the finding might not be national wise representative. Another limitation of this study didn’t assess biochemical markers serum prealbumin due to lack of reagent and financial issue. In addition, 2/3 of the participants were female which is most likely not representative of the population of patients with cancer. Moreover, due to cross-sectional nature of the study, associations can be identified although correlations are not necessarily derived.

## Conclusion

Based on this study, mean level of Serum albumin, TP and Hgb were deranged in malnourished as compared to nourished adult patients with cancer. In addition to nutritional status, type of cancer and stage of cancer are associated with hypoalbuminemia, hypoproteinemia and low Hgb level in adult patients with cancer. Serum albumin and Hgb level were highly correlated with SGA tool but TP is weakly associated with SGA tool. Therefore, this correlation helps to recommend health professionals should preferentially focus on serum albumin and Hgb as alternative diagnostic modality for prompt identification of malnutrition before sign and symptoms manifest and to monitor adult patients with cancer on treatment. We suggest health professional to incorporate serum albumin and Hgb as one of the routine checkup in every cycle of treatment for nutrition counseling.

## Electronic supplementary material

Below is the link to the electronic supplementary material.

Supplementary Material 1

## Data Availability

The datasets used and/or analyzed during the current study are available from the corresponding author on request.

## List of abbreviations

ABP: Albumin Binding Protein
BMI: Body Mass Index
CBC: Complete Blood Count
COVID-19: Corona Virus Diseases − 2019
CRP: C - reactive protein
DDS: Dietary Diversity Score
DM: Diabetes Mellitus
EDTA: Ethylene Diamine Tetra Acetic acid
FAO: Food and Agriculture Organization
GI: Gastro Intestinal
Hgb: Hemoglobin
HIV: Human Immunodeficiency Virus
HTN: Hypertension
Hx: History
IRB: Institutional Review Board
MNA: Mini Nutritional Assesment Tool
MUAC: Mid-Upper Arm Circumference
NRS: Nutrition Risk Screening
NSCLC: Non-Small Cell Lung Cancer
PI: Principal Investigator
ROS: Reactive oxygen species
SGA: Subjective Global Assessment Tool
SOP: Standard Operating Procedures
SPSS: Statistical Package for Social Sciences
SST: Serum Separator Tube
TASH: Tikur Anbessa Specialized Hospital
TNF-α: Tumor Necrosis Factor- alpha
TP: Total Protein
TNM: Tumor Node Metastases
