# Supplementary material for: Blood biomarkers as potential malnutrition screening alternatives among adult patients with cancer on treatment in oncology unit of jimma tertiary hospital: A cross-sectional analysis
Source: BMC Nutr. 2023 Mar 3;9:38. doi: 10.1186/s40795-023-00694-0 (PMC9982783; doi:10.1186/s40795-023-00694-0)
Supplement: Supplementary file 1 — Supplementary Material 1 [file 40795_2023_694_MOESM1_ESM.docx]

SUPPLEMENTARY FILES

**PART IV: SUBJECTIVE GLOBAL ASSESMENT FORM (FIILED BY HEALTH PROFFESIONALS)**

This part of the questionnaire is about the nutritional assessment of the patient. Put circle/tick (√) on the responses of the respondent from the given alternatives.

| **PART IV - SUBJECTIVE GLOBAL ASSESMENT FORM** | | | | |
| --- | --- | --- | --- | --- |
| **S. No** | **Question** | **Response** |  | **Code** |
| **1** | Weight change over 6 months | Weight gain or No change or Mild weight loss  Moderate weight loss  Severe weight loss | **A**  **B**  **C** |  |
| **2** | Weight change in past 2 weeks | Weight is increasing  No change in weight  Weight is decreasing | **A**  **B**  **C** |  |
| 3 | Change in dietary intake | No change or slight change for short duration  Intake borderline and decreasing; Intake poor and increasing; Intake poor, no change based on prior intake  Intake poor and decreasing | **A**  **B**  **C** |  |
| 4 | Duration and degree of change | Less than 2 weeks, little or no change  More than 2 weeks, mild to moderate suboptimal diet  Unable to eat or starvation | **A**  **B**  **C** |  |
| 5 | Presence of GI symptoms | Few or no symptoms intermittently  Some symptoms for >2 weeks; severe symptoms that are improving  Symptoms daily or frequently >2 weeks | **A**  **B**  **C** |  |
| 6 | Functional status | No impairment in strength, stamina and full functional capacity; mild-moderate loss and improving  Mild to moderate loss of strength, stamina / some loss of daily activity or severe loss but now improving  Severe loss of function, stamina and strength | **A**  **B**  **C** |  |
| 7 | Disease state and co-morbidity | No stress  Low or moderate stress  High stress | **A**  **B**  **C** |  |
| 8 | Subcutaneous loss of fat | Little or no loss  Mild-moderate in all areas; severe loss in some areas  Severe loss in most areas | **A**  **B**  **C** |  |
| 9 | Muscle wasting | Little or no loss  Mild to moderate in all areas; severe loss in some areas  Severe loss in most areas | **A**  **B**  **C** |  |
| 10 | Edema | Little or no edema  Mild to moderate edema  Severe edema | **A**  **B**  **C** |  |
| 11 | Ascites | No ascites or only on imaging  Mild to moderate ascites or improving clinically  Severe ascites or progressive ascites | **A**  **B**  **C** |  |

**How to finalize the SGA score**

SGA is truly a subjective means of assessing the nutritional status. SGA classifies the patient as: A. Well-nourished; B. Moderately malnourished; C. Severely malnourished. Patients are placed into one of these categories based on their subjective rating in two broad areas: The items on the form are used by the nutritionist to obtain a general feel for the patient’s status. If there are more B or, the patient is more likely to be moderate malnourished or C ratings , the patients classified as severe malnutrition and score A was well nutrition. If the ratings are on the left-hand side, the patient is likely to be well nourished.
